# Factors Associated with Not Testing For HIV and Consistent Condom Use among Men in Soweto, South Africa

**DOI:** 10.1371/journal.pone.0062637

**Published:** 2013-05-16

**Authors:** Sakhile Mhlongo, Janan Dietrich, Kennedy N. Otwombe, Gavin Robertson, Thomas J. Coates, Glenda Gray

**Affiliations:** 1 Perinatal HIV Research Unit, University of the Witwatersrand, Johannesburg, Gauteng, South Africa; 2 Department of Medicine, University of California Los Angeles, Los Angeles, California, United States of America; 3 Canada-Africa Prevention Trials Network, The Ottawa Hospital General Campus, Ottawa, Ontario, Canada; Fundacion Huesped, Argentina

## Abstract

**Background:**

Besides access to medical male circumcision, HIV testing, access to condoms and consistent condom use are additional strategies men can use to prevent HIV acquisition. We examine male behavior toward testing and condom use.

**Objective:**

To determine factors associated with never testing for HIV and consistent condom use among men who never test in Soweto.

**Methods:**

A cross-sectional survey in Soweto was conducted in 1539 men aged 18–32 years in 2007. Data were collected on socio-demographic and behavioral characteristics to determine factors associated with not testing and consistent condom use.

**Results:**

Over two thirds (71%) of men had not had an HIV test and the majority (55%, n = 602) were young (18–23). Of those not testing, condom use was poor (44%, n = 304). Men who were 18–23 years (aOR: 2.261, CI: 1.534–3.331), with primary (aOR: 2.096, CI: 1.058–4.153) or high school (aOR: 1.622, CI: 1.078–2.439) education, had sex in the last 6 months (aOR: 1.703, CI: 1.055–2.751), and had ≥1 sexual partner (aOR: 1.749, CI: 1.196–2.557) were more likely not to test. Of those reporting condom use (n = 1036, 67%), consistent condom use was 43% (n = 451). HIV testing did not correlate with condom use.

**Conclusion:**

Low rates of both condom use and HIV testing among men in a high HIV prevalence setting are worrisome and indicate an urgent need to develop innovative behavioral strategies to address this shortfall. Condom use is poor in this population whether tested or not tested for HIV, indicating no association between condom use and HIV testing.

## Introduction

After 30 years into a complex epidemic of HIV/AIDS, it is estimated that there are 33 million people living with HIV globally [1]. In South Africa alone, an estimated 5.7 million people are living with HIV. HIV is transmitted predominantly through heterosexual sex in South Africa and over 2 million of those infected are men aged 15 years and older [2], [3]. As has been shown in studies, men are two to three times more likely to transmit HIV to women than women are to men. This could also be attributable to HIV virus concentrations and other sexually transmitted infections [4], [5].

HIV testing is regarded as a priority area in strategies to prevent the spread of HIV and to provide care, support and treatment to people already living with HIV [6], [7]. As part of the South African national strategic plan for HIV and AIDS, South Africa has seen increased efforts to improve the availability and accessibility of HIV testing services [8]. HIV testing, which includes risk reduction counseling, highly influences one's risk perception of acquiring HIV and has shown huge effects on risky behavior change [9]–[11]. In 2010, the HIV Counseling and Testing campaign was launched, aiming to test 15 million South Africans for HIV by mid 2011. One of the objectives for the campaign was to promote the widespread provision and use of condoms. Over 400 million condoms were distributed by the National Department of Health [12]. In 2010/11, an average of 14.5 condoms per male 15 years and older were distributed nationally. This unfortunately has not translated into a reduction in HIV prevalence in South Africa [2].

Even though South Africa has seen improved HIV testing accessibility and condom distribution [13], [14], HIV testing uptake and consistent and correct condom use still remain a challenge particularly amongst men who are generally known for having poor health-seeking behaviors compared to women [15]–[17]. While condom use may differ with age groups, common barriers for condom use include misconceptions about condoms, socio-economic and gender-based factors which may affect women more than men as they have to negotiate condom use with sexual partners [18], [19]. It is estimated that less than one third of adult males over the age of 15 have ever tested in South Africa [2].

This study was conducted as part of a baseline survey for NIMH Project Accept (HPTN 043), a community-based voluntary counseling and testing (VCT) intervention to reduce HIV incidence in populations at risk in Soweto [14]. This study was done as a follow up on a previous study which looked at predictors for HIV testing among males and females in Soweto [20]. The previous study showed that up to 71% of males and 35% of females had never tested for HIV. This study sought to determine the predictors of not testing and condom use in males reporting vaginal sex in Soweto. Further, associations between knowledge of HIV status, condom use, and sexual risk behavior were explored.

## Methods

### Study Design

This baseline household survey was conducted in 2007 and was part of Project Accept, a Phase III community-level randomized controlled study conducted from 2005 to 2011 in five study sites: [14], [21] Baseline assessment methods have been reported in detail in similar Project Accept baseline and VCT studies [20], [22].

### Study Population and Participant Recruitment

A sample of 1539 men aged 18–32 years were recruited in Soweto, a peri-urban African township located 15 km southwest of Johannesburg in Gauteng Province, South Africa. Soweto has an estimated population of at least more than 1.69 million people [23]. Using a household-number database, aerial photography, and household probability sampling technique, households were randomly selected from the household-number database and grouped according to proximity enumeration areas using aerial maps. Selected households within an enumerated area were visited by interview teams throughout the week until the target sample size was reached. Sample size calculations and field identification of households are described in detail in *NIMH Project Accept (HPTN 043) Study Protocol* [24].

### Data Collection Procedures

After establishing contact and receiving permission from the head of the household, eligible household members were listed and one participant randomly selected for participation using the Kish grid method [25]. With permission from the head of the household, the selected member was then approached for consent for participation. Return visits were scheduled for those selected members who were not present at the time of the initial visit. Enumerated household members were eligible to participate if they (1) were aged 18–32 years, (2) had lived in the community at least 4 months in the past year, and (3) had slept regularly in their household at least 2 nights per week. After written informed consent, a 40 minute-long interviewer-administered survey was conducted in a private area of the participant's home.

### Ethics Statement

All participants provided written informed consent to participate in this study. The survey and participant consent procedure were approved by the University of the Witwatersrand Human Research Ethics Committee. Participants were reimbursed with fifty Rands (approximately 6.0 US dollars) for their time.

### Measurement Instrument

Survey questions were designed collaboratively with all sites [26]. With up to 10 different languages spoken in Soweto, English was chosen as the most common language and was used in most of the surveys. Where necessary, survey questions were translated, back translated, and survey interviews conducted in local languages including Zulu, Tswana, and Pedi. The survey assessed demographic characteristics, sexual behaviors, alcohol and substance use, HIV testing history, reasons for not testing, and consistency of condom use in the last six months.

### Measures

All questions on sexual intercourse and condom use were based on sexual activity in the last 6 months.

#### Demographics

Socio-economic status was assessed as “high” if participant's monthly household income was 1,189 US dollars (approximately R10,000) or more; “medium” if it was within 594–1,188 US dollars (approximately R5,000–R9,999); and “low” if was less than 594 US dollars (less than R5,000).

#### Condom use

Condom use in the last 6 months and was defined as (1) consistent, for those that reported using condoms all the time, and (2) inconsistent, for those who reported using condoms rarely, sometimes, or most of the time in their sexual encounters.

#### HIV testing history

Testing history was measured in 2 categories: (1) those that had never tested for HIV, and (2) those that had tested before.

### Statistical Analyses

The primary outcomes were determination of the predictors of (1) not testing for HIV (2) consistent condom use in males in Soweto. Socio-demographic characteristics were examined descriptively for continuous variables and using frequencies for categorical ones. Differences in continuous variables were tested using a two-sample t-test while chi-square analysis was used to compare categorical variables.

Condom use was categorized into two groups: consistent and inconsistent users. These were compared across different socio-demographic variables using chi-square analysis and two-sample t-test for categorical and continuous variables respectively. The proportion of people reporting never testing for HIV was determined using frequencies. Predictors of not testing for HIV and consistent condom use were determined using logistic regression analysis. Model fit was determined by the Hosmer-Lemeshow test. Analysis was performed at a 5% level of significance using SAS 9.2 software.

## Results

**Figure 1:**
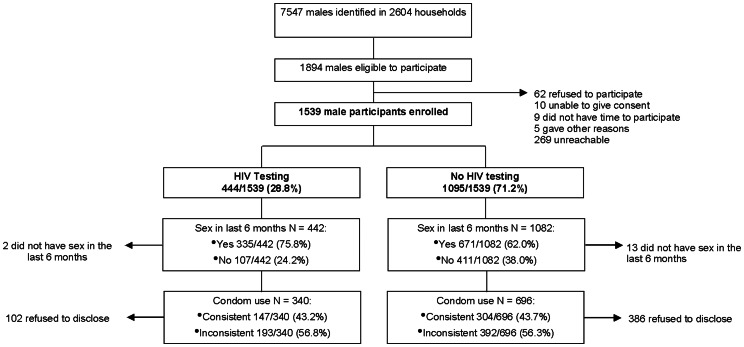
Participant disposition flow diagram.

Figure 1 shows study participant disposition. There were 1539 male participants recruited from a total of 1894 eligible males after visiting 2604 households.

### Demographic Characteristics

The overall median age was 24 (IQR: 21–28) years while the majority (48%, n = 741) of the participants were in the age group 18–23 years. Majority (81%, n = 1243) had secondary education, 52%, (n = 806) were employed, and 85% (n = 694) were in the ‘low income’ group. Only 7% (n = 103) were married (Table 1).

**Table 1:** Socio-demographic characteristics for men reporting ever testing versus those never testing for HIV in Soweto (N = 1539).

Variable	Overall	Ever Tested		*P*-value
		Yes, N (%)	No, N (%)	95% CI
**Total Males enrolled**	**1539**	**444 (28.8%)**	**1095 (71.2%)**	
Median age (IQR) in years	24 (21–28)	26 (23–29)	23 (20–27)	**<.0001**
Age-group (Years)				
18–23	741 (48.2%)	139 (31.3%)	602 (55.0%)	**<.0001**
24–28	479 (31.1%)	182 (41.0%)	297 (27.1%)	
>28	319 (20.7%)	123 (27.7%)	196 (17.9%)	
Education*				
8–12 years	1243 (81.5%)	342 (77.6%)	901 (83.1%)	**<.0001**
≤7 years	114 (7.5%)	26 (5.9%)	88 (8.1%)	
>12 years	168 (11.0%)	73 (16.6%)	95 (8.8%)	
Occupation*				
Employed	806 (52.7%)	295 (66.9%)	511 (47.0%)	**<.0001**
Student	302 (19.8%)	42 (9.5%)	260 (23.9%)	
Unemployed	420 (27.5%)	104 (23.6%)	316 (29.1%)	
Income Group*				
High	33 (4.0%)	15 (5.6%)	18 (3.2%)	**0.0429**
Medium	100 (12.1%)	40 (15.0%)	60 (10.7%)	
Low	694 (84.9%)	212 (79.4%)	482 (86.1%)	
Marital status*				
Married	103 (6.7%)	49 (11.0%)	54 (4.9%)	**<.0001**
Single	1434 (93.3%)	395 (89.0%)	1039 (95.1%)	
Currently have a sex partner*				
No	470 (30.5%)	98 (22.1%)	372 (34.0%)	**<.0001**
Yes	1069 (69.5%)	346 (77.9%)	723 (66.0%)	
Condom use*				
Consistent	451 (43.5%)	147 (43.2%)	304 (43.7%)	0.8926
Inconsistent	585 (56.5%)	193 (56.8%)	392 (56.3%)	
Live with sex partner*				
No	894 (82.8%)	266 (76.4%)	628 (85.8%)	**<.0001**
Yes	186 (17.2%)	82 (23.6%)	104 (14.2%)	
Ever used alcohol*				
No	297 (19.3%)	72 (16.2%)	225 (20.6%)	**0.0493**
Yes	1240 (80.7%)	372 (83.8%)	868 (79.4%)	
Ever used drugs*				
No	1172 (76.3%)	332 (74.8%)	840 (76.9%)	0.3856
Yes	365 (23.7%)	112 (25.2%)	253 (23.1%)	
Ever had vaginal sex*				
No	160 (10.4%)	22 (5.0%)	138 (12.7%)	**<.0001**
Yes	1372 (89.6%)	421 (95.0%)	951 (87.3%)	
Sex in the last six months*				
No	518 (34.0%)	107 (24.2%)	411 (38.0%)	**<.0001**
Yes	1006 (66.0%)	335 (75.8%)	671 (62.0%)	
Talked about HIV in the past 6 months*				
No	192 (14.5%)	49 (11.7%)	143 (15.7%)	0.051
Yes	1135 (85.5%)	370 (88.3%)	765 (84.3%)	
Sex frequency*				
>4 times a week	83 (8.4%)	33 (10.0%)	50 (7.5%)	0.246
2–4 times a week	296 (29.8%)	97 (29.5%)	199 (30.0%)	
1–2 times a month	339 (34.1%)	101 (30.7%)	238 (35.8%)	
>2 times a month	275 (27.7%)	98 (29.8%)	177 (26.7%)	
Number of partners*				
0–1	735 (47.8%)	272 (61.3%)	463 (42.3%)	**<.0001**
>1	804 (52.2%)	172 (38.7%)	632 (57.7%)	
Used alcohol in the last 30 days*				
No	501 (40.8%)	155 (42.1%)	346 (40.2%)	0.5275
Yes	728 (59.2%)	213 (57.9%)	515 (59.8%)	

Bolded findings reflect statistically significant results (*P*<0.05).

* Numbers in strata may differ from total N due to missing values as some participants chose not to answer a question.

### Demographic Characteristics of Never Testing

Table 1 also shows that over two thirds (71%, n = 1095) of men sampled had never tested for HIV and majority were in the younger age-group of 18–23 years. Their median age was 23 (IQR: 20–27) years while most had secondary education (n = 901, 83%). Those who had never tested were younger than those who had ever tested (23 vs. 26, p<0.0001). Majority (53%, n = 576) of men not tested were unemployed, either a student (24%, n = 260) or unemployed (29%, n = 316). Most (86%, n = 482) were in the ‘low income’ group. Of those not testing, 66% (n = 723) had a sex partner, 86% (n = 628) did not live with their sex partner, 84% (n = 765) talked about HIV and 58% (n = 632) had more than one partner. The proportion with secondary education of 8–12 years was significantly higher than those with tertiary or <7 years of education (83%, n = 901; p<0.0001).

### Factors Associated with Never Testing

The unadjusted and adjusted predictors of not testing are presented in Table 2. In the adjusted logistic regression, being in the age-group 18–23 years (aOR: 2.261, CI: 1.534–3.331), not having tertiary education, having had sex in the last 6 months (aOR: 1.703, CI: 1.055–2.751) and having more than one sexual partner (aOR: 1.749, CI: 1.196–2.557) predicted not testing.

**Table 2:** Predictors for never testing for HIV in men in Soweto.

Variable	Unadjusted		Adjusted	
	OR (95% CI)	*P*-value	aOR (95% (CI)	*P*-value
Age-Group (Years)				
18–23	**2.718 (2.031–3.637)**	**<.0001**	**2.261 (1.534–3.331)**	**<.0001**
24–28	1.024 (0.765–1.370)	0.8728	1.098 (0.761–1.584)	0.6169
>28	Ref		Ref	
Education (Years)				
8–12	**2.025 (1.457–2.815)**	**<.0001**	**1.622 (1.078–2.439)**	**0.0202**
≤7	**2.601 (1.526–4.434)**	**0.0004**	**2.096 (1.058–4.153)**	**0.034**
>12	Ref		Ref	
Occupation				
Employed	Ref			
Student	**3.574 (2.503–5.102)**	**<.0001**		
Unemployed	**1.754 (1.347–2.284)**	**<.0001**		
Income Group				
High	0.528 (0.261–1.067)	0.0752		
Medium	0.660 (0.429–1.016)	0.0588		
Low	Ref			
Marital status				
Married	Ref		Ref	
Single	**2.387 (1.594–3.574)**	**<.0001**	1.082 (0.624–1.876)	0.7794
Currently have a sex partner				
Yes	Ref		Ref	
No	**1.817 (1.405–2.348)**	**<.0001**	1.725 (0.461–6.453)	0.418
Condom use in the past 6 months				
Consistent	1.018 (0.784–1.323)	0.8927		
Inconsistent	Ref			
Live with sex partner				
Yes	Ref		Ref	
No	**1.862 (1.348–2.571)**	**0.0002**	1.244 (0.802–1.931)	0.3303
Ever used alcohol				
Yes	Ref		Ref	
No	**1.339 (1.000–1.793)**	**0.0498**	1.117 (0.760–1.641)	0.5746
Ever used drugs				
Yes	Ref			
No	1.120 (0.867–1.447)	0.3857		
Ever had vaginal sex				
Yes	Ref			
No	**2.777 (1.745–4.418)**	**<.0001**		
Sex in the last 6 months				
Yes	**0.521 (0.406–0.669)**	**<.0001**	**1.703 (1.055–2.751)**	**0.0295**
No	Ref		Ref	
Talked about HIV/AIDS in past 6 months				
Yes	Ref		Ref	
No	1.411 (0.997–1.998)	0.0519	1.007 (0.653–1.551)	0.9764
Frequency of sex in the last 6 months				
>4 times a week	1.354 (0.820–2.237)	0.2368		
2–4 times a week	Ref			
1–2 times a month	1.555 (0.946–2.558)	0.0818		
>2 times a month	1.192 (0.720–1.973)	0.4946		
Number of sex partners				
0–1	Ref		Ref	
>1	**2.159 (1.723–2.705)**	**<.0001**	**1.749 (1.196–2.557)**	**0.004**
Used alcohol in the last 30 days				
Yes	Ref			
No	1.038 (0.833–1.295)	0.7375		

Bolded findings reflect statistically significant results (*P*<0.05).

### Demographic Characteristics of Condom Use

Among males who reported never testing (Table 3), data was available for 696 participants. Of the remaining 399, 386 did not have sex in the last 6 months and 13 chose not to answer (Figure 1). The number using condoms consistently was higher in the age-group 18–23 years (n = 184). The number of inconsistent condom users was significantly higher than the consistent users (392 vs. 304, p<0.0001). The number of employed males was significantly higher in the inconsistent compared with the consistent (233/387 vs. 143/304; p = 0.0006). There was no association between condom use and talking about HIV (p = 0.1904). The proportion of males with more than one sexual partner in the inconsistent group was significantly higher than the consistent (104/203 vs. 98/304; p<0.0001).

**Table 3:** Socio-demographic characteristics for males who never tested for HIV by condom use in Soweto (N = 696).

Variable	Consistent condom users, N (%)	Inconsistent condom users, N (%)	*P*-value
	304 (40.8%)	392 (59.2%)	
Age group (Years)			
18–23	184 (60.5%)	144 (36.7%)	**<.0001**
24–28	75 (24.7%)	153 (39.0%)	
>28	45 (14.8%)	95 (24.2%)	
Education (Years)*			
8–12	255 (84.4%)	310 (79.9%)	**0.0032**
≤7	12 (4.0%)	42 (10.8%)	
>12	35 (11.6%)	36 (9.3%)	
Occupation*			
Employed	143 (47.0%)	233 (60.2%)	**0.0003**
Student	62 (20.4%)	43 911.1%)	
Unemployed	99 (32.6%)	111 (28.7%)	
Income group*			
High	6 (4.2%)	8 (3.6%)	-
Low	122 (84.7%)	186 (84.5%)	
Medium	16 (11.1%)	26 (11.8%)	
Marital status*			
Married	5 (1.6%)	44 (11.3%)	-
Single	299 (98.4%)	346 (88.7%)	
Currently have a sex partner			
No	61 (20.1%)	29 (7.4%)	**<.0001**
Yes	243 (79.9%)	363 (92.6%)	
Live with sex partner*			
No	230 (95.4%)	272 (75.1%)	**<.0001**
Yes	11 (4.6%)	90 (24.9%)	
Ever used alcohol*			
No	49 (16.1%)	33 (16.3%)	0.751
Yes	255 (83.9%)	169 (83.7%)	
Ever used drugs*			
No	225 (74.0%)	154 (76.2%)	0.825
Yes	79 (26.0%)	48 (23.8%)	
Ever had vaginal sex*			
No	1 (0.3%)	8 (4.1%)	-
Yes	303 (99.7%)	189 (95.9%)	
Sex in the last six months*			
No	0 (0.0%)	12 (6.3%)	-
Yes	304 (100%)	178 (93.7%)	
Talked about HIV in the past 6 months*			
No	30 (11.4%)	21 (12.1%)	0.1904
Yes	233 (88.6%)	152 (87.9%)	
Sex frequency*			
>4 times a week	13 (4.3%)	15 (8.5%)	**<.0001**
2–4 times a week	73 (24.4%)	61 (34.5%)	
1–2 times a month	135 (45.2%)	54 (30.5%)	
>2 times a month	78 (26.1%)	47 (26.6%)	
Number of partners*			
0–1	206 (67.8%)	99 (48.8%)	**<.0001**
>1	98 (32.2%)	104 (51.2%)	
Used alcohol in the last 30 days*			
No	97 (38.5%)	50 (29.8%)	0.1529
Yes	155 (61.5%)	118 (70.2%)	

Bolded findings reflect statistically significant results (*P*<0.05).

* Numbers in strata may differ from total N due to missing values as some participants chose not to answer a question.

### Factors Associated with Condom Use

In the adjusted logistic regression in Table 4, being in the age-group 18–23 years (OR: 1.827, CI: 1.023–3.263), having 12 years or more of education (aOR: 3.077, CI: 1.085–8.729), not living with a sex partner (aOR: 4.117, CI: 1.911–8.870), and having talked about HIV in the past 6 months prior to this study (aOR: 1.819, CI: 1.004–3.294) predicted consistent condom use.

**Table 4:** Predictors of consistent condom use in males who never tested for HIV in Soweto.

Variable	Unadjusted		Adjusted	
	OR (CI)	*P*-value	aOR (CI)	*P*-value
Age group (Years)				
18–23	**2.697 (1.779–4.090)**	**<.0001**	**1.827 (1.023–3.263)**	**0.0417**
24–28	1.035 (0.660–1.622)	0.8814	1.070 (0.602–1.902)	0.8176
>28				
Education (Years)				
8–12	**2.879 (1.484–5.585)**	**0.0018**	2.095 (0.833–5.265)	0.1159
<7	Ref		Ref	
>12	**3.403 (1.540–7.516)**	**0.0025**	**3.077 (1.085–8.729)**	**0.0346**
Occupation				
Employed	Ref		Ref	
Student	**2.349 (1.511–3.652)**	**<0.0001**	1.336 (0.732–2.437)	0.3448
Unemployed	**1.453 (1.033–2.045)**	**0.032**	1.172 (0.745–1.844)	0.4924
Income Group				
High	1.143 (0.387–3.377)	0.8083		-
Medium	0.938 (0.483–1.821)	0.8505		
Low	Ref			
Marital status				
Married	Ref		Ref	
Single	**7.605 (2.977–19.43)**	**<.0001**	1.745 (0.579–5.255)	0.3225
Currently have a sex partner				
Yes	Ref			
No	**3.142 (1.962–5.032)**	**<.0001**		-
Live with sex partner				
Yes	Ref		Ref	
No	**6.918 (3.611–13.25)**	**<.0001**	**4.117 (1.911–8.870)**	**0.0003**
Ever used alcohol				
Yes	1.099 (0.735–1.643)	0.6456		
No	Ref			
Ever used drugs				
Yes	1.061 (0.752–1.496)	0.7372		-
No	Ref			
Ever had vaginal sex				
Yes	6.413 (0.798–51.55)	0.0806	0.880 (0.051–15.24)	0.9302
No	Ref		Ref	
Talked about HIV/AIDS in past 6 months				
Yes	1.331 (0.817–2.169)	0.2504	**1.819 (1.004–3.294)**	**0.0484**
No	0.751 (0.461–1.224)	0.2504	Ref	
Frequency of sex in the last 6 months				
>4 times a week	0.606 (0.303–1.215)	0.1581		-
2–4 times a week	Ref			
1–2 times a month	**2.262 (1.538–3.327)**	**<.0001**		
>2 times a month	1.360 (0.899–2.057)	0.1453		
Number of sex partners				
0–1	Ref			-
>1	0.906 (0.659–1.245)	0.5416		
Used alcohol in the last 30 days				
Yes	Ref			-
No	1.144 (0.847–1.544)	0.38		

Bolded findings reflect statistically significant results (*P*<0.05).

## Discussion

Our study consisted of predominantly black, low income male participants aged 18–32 years, which was representative of a male population with high HIV prevalence in South Africa [27], [28]. The findings from this study show that men 23 years or younger do not generally test for HIV but use condoms more as compared to older men. There was however no overall association between not testing and condom use.

Despite a high burden of HIV in Soweto, more than two thirds of men had never tested for HIV. Being unaware of one's HIV status is a concern given the widespread and the various national and international initiatives using HIV testing as the cornerstone of HIV prevention strategies [29], [30]. However, it is easy to assume that not knowing one's status may influence the decision to use condoms more regularly especially among single men. This study provides important insights about HIV testing and condom use behaviors amongst males living in a poor-resourced and high HIV prevalence setting. We show that younger males who are sexually active and who have multiple partners are less likely to test for HIV but are more likely to use condoms consistently. Conversely, older males may be more likely to test for HIV but continue to engage in higher risk sexual behaviors through multiple sexual partners and inconsistent condom use. Several studies that investigate condom use and uptake of HIV testing [9], [16], [20], [31], [32] have been conducted in similar HIV hyper-endemic settings but few if any have looked at predictors of not testing and consistency of condom use at the same time for those who have tested and those who have never tested for HIV before.

Knowledge of HIV status particularly amongst men is a critical step in HIV prevention as it has been linked to decreased risky behavior for those who test positive for HIV [33], [34]. Our data show a strong association between low education level and unemployment with not testing. This may suggest that literacy may be among other things a barrier for accessing VCT in this kind of setting. Therefore, as VCT service roll-out increases to cope with HIV incidence reduction demand in South Africa, more alternative VCT models will be required to improve the uptake in hard-to-reach resource-limited populations.

Risk reduction still remains one of the key areas in HIV prevention in South Africa as over half of our sample reported having more than one sexual partner and at the same time showing higher likelihood of not testing. VCT is a proper platform to address risk behavior during testing.

Another issue of concern in this population is that less than half (43%) reported consistent condom use and these were mostly single men. During this period, the national HIV incidence survey had reported high incidence rates in this age group. Also, in line with the national HIV incidence survey findings was condom use, which was generally higher in younger people aged 18–23 years while HIV testing was lower in the same group [2]. Results from a previous study on intentions for condom use among youth suggest that the understanding of the effectiveness of condoms in the prevention of HIV/AIDS and STIs and unwanted pregnancy is likely to be associated with positive attitudes towards condom use [35]. This may suggest a slightly different approach regarding condom education as condom use may not seem appealing or even not relevant in some cases to those in more stable relationships, particularly older age groups and those that live with their sexual partners.

One of Project Accept's aims was to encourage discussions around HIV at both community and family levels. This was to be achieved through open community meetings and also private VCT sessions. Our data showed a reasonable association between consistent condom use and talking about HIV. This may suggest a need for an open community-based approach towards HIV education.

In sub-Saharan Africa, it has been estimated that nearly 80% of HIV-infected adults are unaware of their HIV status [36]. A limitation to our study may be the lack of HIV data from our study participants. Comparing behavioral characteristics against HIV status and condom use would have provided a more accurate assessment of this population. Another limitation to the study could be that the participants were not asked directly about their sexual orientation. So, a potential bias could be the assumption that all participants were heterosexual because they reported vaginal sex in the last six months.

This study has shown low levels of HIV testing and poor condom use in a high-risk population among young single men. It has also shown a lack of association between knowledge of HIV status and condom use. However, it still provides a justifiable platform for NIMH Project Accept (HPTN 043) whose primary aim was to reduce HIV incidence using a community-based VCT model where mobile VCT stations were set up at specific locations in the communities and residents would come and test at their convenience generating a culture and attitude of openness about HIV risk reduction. This may suggest an alternative HIV prevention model from a health care point of view to suit low income populations that experience various barriers to public sector services. A recommendation to the model would be to begin with active community mobilization by emphasizing not only the knowledge of one's HIV status but also risk reduction. Secondly, mobile VCT in communities to encourage hard-to-reach groups like men and youth to take VCT and perhaps re-look condom use education to encourage consistent use among those with risky behaviors such as having multiple sexual partners.
